# Immunometabolic reprogramming in rheumatoid arthritis: the epitranscriptomic role of N6-methyladenosine in innate and adaptive immunity

**DOI:** 10.3389/fimmu.2026.1834919

**Published:** 2026-05-13

**Authors:** Jin Yang, Lei Wan, Hongbo Chen, Shu Li, Yuwei Zhou

**Affiliations:** 1 The First Affiliated Hospital of Anhui University of Chinese Medicine, Hefei, Anhui, China; 2 Anhui University of Chinese Medicine First Clinical Medical College, Hefei, Anhui, China

**Keywords:** epigenetics, m6A methylation, metabolic reprogramming, rheumatoid arthritis, synergistic regulation

## Abstract

N6-methyladenosine (m6A) methylation is the most common intramolecular modification in eukaryotic mRNA; its dynamic regulation depends on “writers” (methyltransferases: METTL3/METTL14/WTAP/VIRMA), “erasers” (demethylases: FTO/ALKBH5), and “readers” (binding proteins: YTHDF/YTHDC/IGF2BP families), thereby regulating RNA splicing, nuclear export, translation, and degradation. In rheumatoid arthritis (RA), this epigenetic network is severely disrupted: abnormal expression of writers leads to post-transcriptional activation of pro-inflammatory genes, while an imbalance in erasers compromises the stability of mRNAs encoding key signaling molecules. Together, these factors promote abnormal differentiation of immune cells, invasive proliferation of fibroblast-like synovial cells, and cartilage erosion. At the same time, hypoxia, inflammatory cytokines, and metabolic stress present in the joint microenvironment of RA induce cellular metabolic reprogramming, characterized by a shift toward aerobic glycolysis (Warburg effect), a reorganization of lipid synthesis and oxidation pathways, and an increase in glutamine uptake and catabolism; these changes all contribute to accelerating disease progression. Recent data have revealed a foundational integration between m6A modification and metabolic reprogramming: m6A regulators directly reshape the metabolic network by targeting transcripts encoding the glycolysis-limiting enzyme (HK2), key molecules in lipid metabolism (FASN/CPT1), and amino acid transporters (SLC1A5), thereby coordinating immune inflammation and tissue destruction in RA. This review elucidates the regulatory role of m6A methylation in the metabolic reprogramming of RA and explains how writers, erasers, and readers influence disease progression by participating in glycolysis, lipid metabolism, and glutamine metabolism. By focusing on the central question of whether m6A modification is the root cause of metabolic reprogramming in the pathogenesis of RA, we have integrated existing data to define the “m6A-metabolism-immunity” regulatory axis and identified potential therapeutic strategies targeting this association.

## 1 Introduction

Rheumatoid arthritis has long been defined by its immunologic and genetic underpinnings. Yet a fundamental question remains unresolved: how do chronically activated immune and stromal cells sustain their relentless effector functions in the face of profound metabolic stress? This review proposes that the answer lies at the nexus of two revolutionary fields, epitranscriptomics and immunometabolism, and that N6-methyladenosine (m6A) RNA methylation acts as the master regulator of this pathogenic alliance. By framing this interplay as an integrated “m6A-metabolism-immunity axis,” we challenge the conventional view that metabolic reprogramming is merely a downstream consequence of inflammation, and instead posit that m6A-driven metabolic rewiring is a root cause of disease chronicity and therapeutic resistance. RA is a chronic autoimmune-mediated disease that is modulated by various immune cells and signaling networks (1), affecting approximately 1% of the global population (2, 3). In genetically predisposed individuals, environmental factors such as smoking or infections can promote the synthesis of autoantibodies, including ACPA and rheumatoid factor. The resultant immune complexes stimulate synovitis, as Th1/Th17 cells, macrophages, and B cells secrete pro-inflammatory mediators (e.g., TNF-α, IL-6, IL-17, RANKL), while synovial fibroblasts exacerbate the condition by producing VEGF and MMPs. This promotes the distinctive clinical features of RA, including joint damage, synovial hyperplasia, and extra-articular complications such as pulmonary diseases (4, 5). Despite the known involvement of genetic and environmental factors in RA etiology, the precise underlying molecular mechanisms remain largely elusive (6–8). Recently, the dysregulation of N6-methyladenosine (m6A) methylation and metabolic reprogramming has emerged as a central driver to RA pathogenesis (9). m6A is the primary epigenetic modification of eukaryotic mRNA and forms a dynamic network comprising writers (methyltransferases: METTL3, METTL14, WTAP) (10), erasers (demethylases: FTO, ALKBH5), and readers (binding proteins: YTHDF, YTHDC families), which modulate all RNA processes (11, 12). This regulatory network is significantly disrupted in RA. For instance, METTL3 expression is elevated in synovial cells, which exacerbates inflammation, while erasers such as FTO demonstrate distinct correlations with disease activity and joint destruction (13, 14). Metabolic reprogramming is a pivotal mechanism allowing RA effector cells to adapt to the hostile inflammatory microenvironment. This process involves several key pathways: glucose metabolism (characterized by Warburg effect-driven glycolysis), lipid metabolism (governed by FASN-promoted synthesis and CPT1-facilitated β-oxidation), and amino acid metabolism (predominantly SLC1A5-mediated glutamine uptake) (15, 16). Moreover, several studies have indicated that m6A directly regulates metabolic reprogramming by targeting key metabolic mRNAs. For instance, m6A modification of HK2 mRNA has been shown to promote glycolysis (17). The central question addressed by this review is whether m6A methylation contributes to RA pathogenesis by orchestrating metabolic reprogramming in key effector cells. We synthesize current evidence on the regulatory mechanisms of m6A modification, its pathogenic role in RA, and its impact on metabolic pathways, with an emphasis on the causal links between epitranscriptomic changes and metabolic alterations. By framing this interplay as an integrated “m6A-metabolism-immunity axis,” we aim to provide a conceptual framework for future research and the identification of novel therapeutic targets.

## 2 m6A methylation modification

The m6A modification—the most prevalent internal chemical modification in eukaryotic mRNA—is dynamically regulated by a dedicated set of proteins classified as “Writers” (methyltransferases), “Erasers” (demethylases), and “Readers” (effector proteins). **Table 1** summarizes this core machinery.

**Table 1:** Comprehensive overview of m6A RNA methylation regulatory machinery.

Type	Factor	Full name	Function
m6A Writers	METTL3	Methyltransferase-like 3	Catalyzes m6A methylation (installation) on target mRNAs
	METTL14	Methyltransferase-like 14	Serves as a structural scaffold for METTL3 to mediate m6A methylation.
	WTAP	Wilms tumor 1- associated protein	Facilitates the localization of the methyltransferase complex to nuclear speckles.
	VIRMA/KIAA1429	Vir-like m6A methyltransferase associated	Recruits the writer complex to guide m6A methylation at specific mRNA sites.
m6A Erasers	FTO	Fat mass and obesity-associated	Mediates m6A demethylation to regulate mRNA splicing and translation.
	ALKBH5	AlkB homologue 5	Catalyzes m6A demethylation to facilitate mRNA nuclear export and processing.
m6A Readers	YTHDF1	YTH N6-methyladenosine RNA binding protein 1	Recognizes m6A sites to promote mRNA translation initiation.
	YTHDF2	YTH N6-methyladenosine RNA binding protein 2	Recognizes m6A sites to trigger mRNA degradation.
	YTHDF3	YTH N6-methyladenosine RNA binding protein 3	Coordinates with YTHDF1/2 to modulate translation and decay.
	YTHDC1	YTH domain containing 1	Regulates mRNA alternative splicing and nuclear export.
	YTHDC2	YTH domain containing 2	Enhances translation efficiency and regulates mRNA stability.

### 2.1 m6A writers(methyl transferases)

The m6A Methyl Transferase Complex (MTC) is the primary regulator of RNA m6A modification, mainly comprising methyltransferases (METTL3, METTL14, WTAP, and VIRMA) (18–20). METTL3 is the only catalytic subunit with methyltransferase activity and is the main enzyme of MTC. Furthermore, this catalytic core has been comprehensively studied in structural biology (21), which showed that METTL3 and METTL14 form a stable heterodimer, which constitutes the structural and functional unit for m6A deposition (22). Moreover, METTL3 contains the catalytic site, while METTL14, despite lacking catalytic activity, is crucially involved in stabilizing METTL3 and recognizing the RNA substrate, thus ensuring precise RNA binding and methylation. The absence of METTL14 can significantly reduce intracellular m6A modification levels (23). WTAP also lacks catalytic activity and functions as a localization and functional guidance subunit, modulating the stability and localization of METTL3 and METTL14 (24). VIRMA is the largest component by molecular weight in MTC and is the RNA-targeting and scaffold subunit (25, 26), which promotes the complex’s targeted binding to specific RNA regions (**Table 1**).

### 2.2 m6A erasers (demethylases)

m6A Erasers mediate the dynamic and reversible regulation of m6A methylation through the process of demethylation (14). Currently, the reported core enzymes include only FTO and ALKBH5 (**Table 1**). FTO was the first identified m6A eraser and is localized in both the cytoplasm and the nucleus (27). Functionally, FTO predominantly catalyzes m6A demethylation on precursor mRNAs to modulate selective splicing and 3’-UTR processing, thereby regulating energy metabolism (28, 29). FTO expression has been significantly associated with human weight gain and lipid metabolic homeostasis (30). ALKBH5, another key member of the AlkB protein family, is primarily localized in the nucleus. Its expression level negatively correlates with global mRNA m6A levels. As a specific eraser, ALKBH5 alters the m6A landscape via direct demethylation, which subsequently promotes the assembly of mRNA processing factors and facilitates mRNA nuclear export. The discovery and characterization of these two erasers have validated that m6A modification is a reversible and highly regulated epigenetic process, providing a foundation for understanding the plasticity of the epitranscriptome.

### 2.3 m6A reader (RNA-binding proteins)

Reader proteins are the key mediators in the m6A methylation process and mainly include the proteins from the YTH m6A RNA-binding protein (YTHDF) and insulin-like growth factor 2 mRNA-binding protein (IGF2BP) families (31). In human cells, YTH domain (YTHDF1–3 and YTHDC1-2) containing proteins specifically recognize and bind m6A-modified RNA, thus modulating mRNA splicing, nuclear export, translation, and degradation, as well as their efficiencies (10, 32). Furthermore, YTHDF1 binds m6A-modified RNA comprising the RRACH sequence, increasing mRNA translation efficiency. YTHDF2 recognizes m6A-modified RNA but mainly modulates mRNA degradation (33). YTHDF3 assists both YTHDF1 and YTHDF2 to synergistically regulate mRNA translation and degradation (34). YTHDC1 is localized in the nucleus, where it interacts with m6A-modified RNA to regulate alternative splicing and facilitate nuclear export. YTHDC2 predominantly exists in the cytoplasm, identifying m6A alterations to regulate mRNA translation efficiency and degradation (35). The IGF2BP family represents a unique class of m6A-reading proteins (**Table 1**). The three IGF2BP (IGF2BP1-3) members have similar functions, which depend on the presence of m6A methylation. These proteins improve mRNA stability by recognizing m6A methylation.

## 3 Core characteristics of metabolic reprogramming

Metabolic reprogramming is a fundamental adaptive process in which cells modify their metabolic pathways to meet the increased energetic and biosynthetic demands associated with rapid proliferation and immune activation. This reprogramming is not random but converges on several core, interconnected hallmarks across major nutrient classes. **Table 2** summarizes these essential hallmarks, their key regulators, and functional outcomes, which are elaborated in the subsequent sections.

**Table 2:** Essential hallmarks of metabolic reprogramming-the concise version.

Type	Hallmark	Key regulators	Functional outcome
Glucose Reprogramming	Preferential aerobic glycolysis (Warburg effect)	AMPK, MYC, Histone acetylation	Rapid ATP and biosynthetic precursors
Lipid Reprogramming	Enhanced fatty acid synthesis & β-oxidation	FASN, CPT1	Membrane building blocks, alternative energy
Amino Acid Reprogramming	Enhanced glutamine uptake & metabolism, anaplerosis	Glutaminase	Energy, TCA cycle replenishment, biomass synthesis

### 3.1 Glucose metabolic reprogramming

Glucose metabolic reprogramming is the most comprehensively studied type of metabolic reprogramming (16). It encompasses the dynamic remodeling of glucose metabolic pathways by cells in response to physiological or pathological stimuli, disrupting the traditional balance wherein aerobic conditions promote oxidative phosphorylation and anaerobic conditions depend on glycolysis (36). This process adjusts to energy supply and material synthesis requirements by modulating the activity of key enzymes and selecting pathways. Its characteristic feature is the “Warburg effect”, where cells preferentially activate glycolysis even under oxygen-rich conditions (37). Glucose is enzymatically converted to pyruvate, which then transforms into lactate, while the activity of enzymes associated with oxidative phosphorylation is downregulated (38). Because of its high reaction rate, glycolysis offers a rapid energy supply. Its intermediate products, such as nucleotides and fatty acids, are the building blocks for cell proliferation or activation (**Table 2**). Glucose metabolism reprogramming involves signaling pathways, including AMPK and MYC, as well as epigenetic modifications such as histone acetylation (39, 40). This represents a core mechanism for cellular signal integration and functional adaptation.

### 3.2 Lipid metabolic reprogramming

Lipid metabolic reprogramming depends on adaptive alterations to cellular lipid synthesis, degradation, or transport. This process is significantly observed in tumors and inflammatory diseases, and is functionally modulated by targeting lipid metabolic networks (41). It has been observed that for supplying raw materials to rapidly proliferating cell membranes, tumor cells significantly enhance fatty acid synthesis to produce phospholipids, triglycerides, and other lipids (42). Moreover, under glucose-limited conditions, fatty acid β-oxidation acts as an alternative energy pathway to sustain ATP production. Studies have also indicated that in inflammatory states, M1 macrophages also modulate lipid metabolism (36) by increasing lipid breakdown to release energy while generating inflammatory mediator precursors (43). This process depends on the ability of the key enzymes, such as fatty acid synthase (FASN) and carnitine palmitoyl transferase 1 (CPT1), to adapt to cellular functional demands (**Table 2**). Lipid metabolic reprogramming modulates cellular survival and proliferation, providing crucial metabolic support for inflammatory development and tumor progression (44).

### 3.3 Amino acid metabolic reprogramming

Amino acid metabolic reprogramming promotes the dynamic modulation of cellular amino acid uptake, catabolism, and biosynthesis. Glutamine metabolic reprogramming is the most common amino acid metabolic pathway (45). Glutamine serves as a critical anaplerotic substrate for RA cells, fueling the TCA cycle via α-ketoglutarate (α-KG) generation to support ATP production and biosynthesis (46–48). Upregulated glutamine transporters (e.g., SLC1A5) and glutaminase activity ensure sufficient carbon and nitrogen supply for cell proliferation, redox homeostasis (via glutathione synthesis), and inflammatory mediator production (**Table 2**). Undegraded glutamine functions as a fundamental carbon and nitrogen source, facilitating the production of biomolecules like nucleotides, proteins, and glutathione. This supports the energy- and resource-demanding cellular activities such as rapid proliferation and the production of immune effector molecules. Studies have shown that amino acid metabolic reprogramming provides energy and supplies biosynthetic precursors by regulating essential pathways such as glutamine metabolism, meeting specific functional requirements (49).

## 4 Mechanism of m6A methylation in RA

### 4.1 m6A writers (methyltransferases)

**Figure 1:**
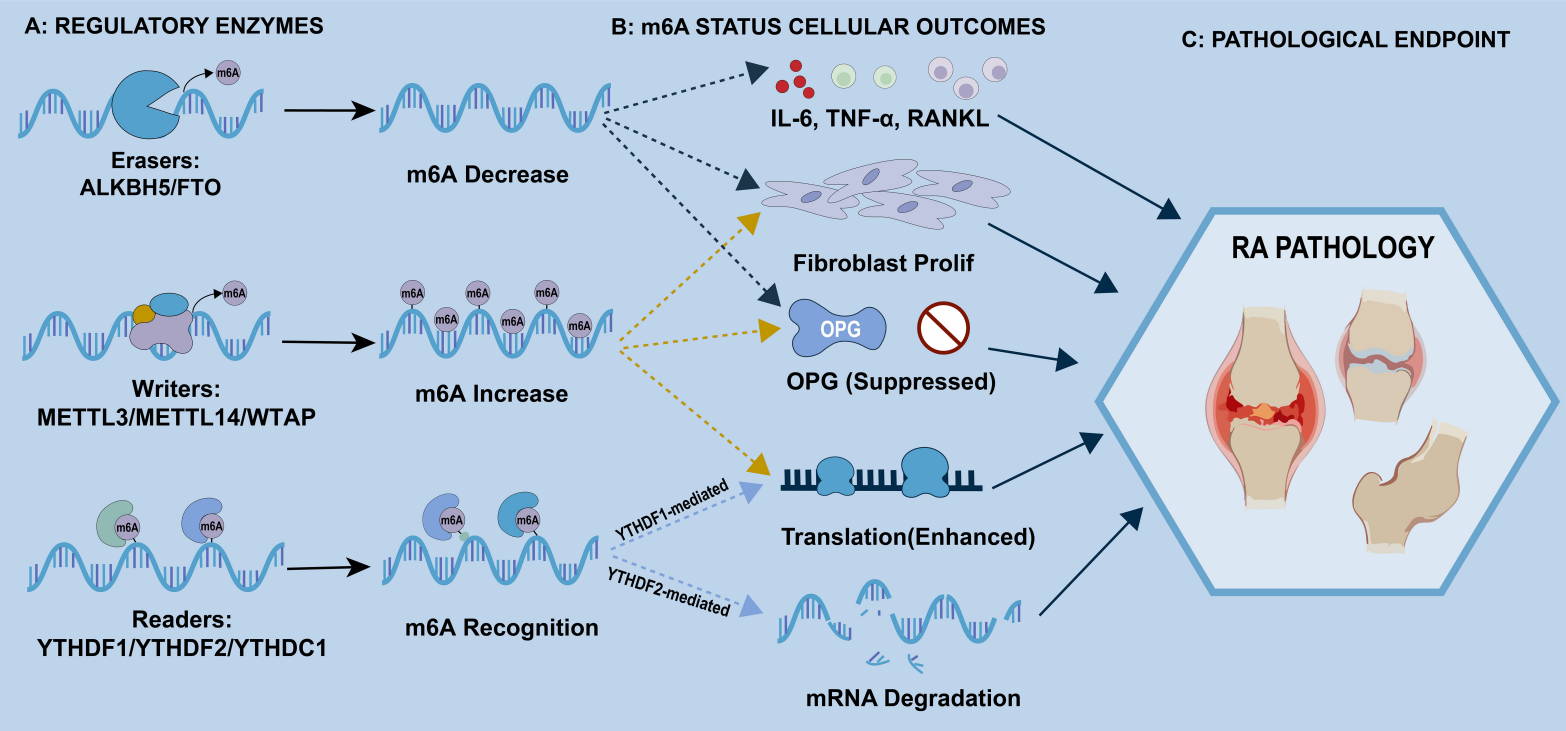
A schematic view of m6A regulation in rheumatoid arthritis pathology. In this model, m6A methylation is deposited by the writer complex (METTL3/METTL14/WTAP) and removed by erasers ALKBH5 and FTO, causing mRNA m6A levels to rise or fall accordingly. The fate of these methylated transcripts depends on which reader proteins bind them: YTHDF1 tends to boost translation, whereas YTHDF2 often triggers mRNA decay. In the RA setting, shifts in m6A status tilt the balance toward higher expression of IL-6, TNF-α, and RANKL, while OPG is suppressed. These changes help fuel fibroblast proliferation and drive disease progression.

METTL3 is significantly upregulated in RA synovial tissues compared with healthy controls (50, 51), and its inhibition attenuates proinflammatory cytokine production (IL-6, TNF-α), synovial hyperplasia, and cartilage erosion (**Figure 1**). METTL3 promotes FLS activation and inflammation, at least in part, through the NF-κB signaling pathway, thereby linking its upregulation to RA joint damage (52). Beyond this generalized inflammatory signaling, METTL3 directly installs m6A marks on mRNAs encoding key metabolic regulators in FLS and activated T cells. These regulators include the glycolytic rate-limiting enzyme HK2 and the amino acid transporter SLC1A5. This epitranscriptomic tagging enhances the translation efficiency of these transcripts, thereby actively driving the Warburg effect and glutaminolysis required for rapid cellular proliferation and sustained inflammatory effector function. This establishes METTL3 as a key driver of inflammation and joint damage in RA by modulating the m6A modification of target RNAs. Moreover, METTL14 expression positively correlates with METTL3, and despite its weaker catalytic activity, METTL14 enhances MTC activity by stabilizing the METTL3 protein structure, thus regulating abnormal FLS proliferation (53). Functionally, METTL14 facilitates the targeted methylation of lipid synthesis genes such as FASN, promoting *de novo* lipogenesis that supplies membrane phospholipids for hyperplastic FLS expansion. Several studies have indicated that METTL14 downregulation inhibits the cyclic progression of synovial fibroblasts and reduces extracellular matrix-degrading enzyme MMP-9 secretion, thus mitigating the articular cartilage damage (54).

In RA patients, WTAP is highly expressed in CD4^+^ T cells, and its expression level is positively correlated with RA disease activity. WTAP promotes T cell-mediated immunological responses by increasing the development of CD4^+^ T cells into Th17 cells, thus serving as an essential regulator of immune dysregulation in RA (55). Mechanistically, WTAP facilitates the installation of m6A marks on transcripts encoding Th17-polarizing regulators, a modification that alters the nuclear export and splicing efficiency of these mRNAs to favor pathogenic Th17 lineage commitment. VIRMA expression demonstrates tissue specificity: its decreased expression in RA chondrocytes correlates with cartilage degradation, whereas its elevated expression in synovial tissue is linked with synovial hyperplasia (56). Previous research suggests that the m6A methyltransferase family regulates RA pathological progression through synergistic or specific effects on different pathways, such as immune activation, synovial proliferation, and cartilage protection, representing a key upstream link in m6A modification in RA pathogenesis (9).

### 4.2 m6A erasers: FTO and ALKBH5

FTO and ALKBH5 exhibit tissue- and disease-specific expression patterns (57). In RA pathogenesis, they play distinct and contrasting roles (**Figure 1**). Molecular analysis of RA patients’ synovial tissue showed a high expression of FTO. Furthermore, Pearson or Spearman correlation analysis confirmed a statistically significant positive correlation between FTO levels and RA progression. FTO increases the stability and translation of the mRNA of proinflammatory factors (IL-17 and IL-23) through demethylation, thus exacerbating synovial inflammation (12); mechanistically, the primary functional consequence of FTO-mediated demethylation is the protection of target transcripts from YTHDF2-mediated decay—by erasing m6A marks, FTO extends the half-life of mRNAs encoding hypoxia-inducible factor (HIF-1α) and glycolytic enzymes (e.g., PFK1, LDHA), thereby sustaining the elevated glycolytic flux characteristic of inflamed RA synovium. Similarly, FTO modulates RNA associated with glycogen metabolism, enhancing glycolytic activity to facilitate aberrant proliferation and the production of matrix metalloproteinase (MMP-13), therefore increasing cartilage erosion (58). Animal studies have confirmed that in collagen-induced arthritis (CIA) mouse models, FTO downregulation significantly reduces joint cavity inflammatory factors, synovial hyperplasia, and bone destruction (27).

In RA, ALKBH5 exhibits diverse beneficial roles, compared to FTO’s proinflammatory effect. It is highly expressed in chondrocytes and promotes cartilage matrix gene expression through mRNA demethylation (59). In M1 macrophages, ALKBH5 removes m6A modifications from carnitine palmitoyltransferase 1A (CPT1A) mRNA, thereby protecting this transcript from degradation and sustaining fatty acid β-oxidation to meet the bioenergetic demands of localized immune responses. Furthermore, its deficiency in synovial tissues exacerbates pathology by restoring NLRP3 within the ALKBH5-YTHDF2-NLRP3 axis (60). Furthermore, ALKBH5 modulates immune regulation by increasing anti-inflammatory IL-10 expression in innate immunity and suppressing Th17 polarization to rebalance the Treg/Th17 axis in adaptive immunity (61, 62). This tissue-specific expression pattern and functional dichotomy extend beyond RA to other pathologies, such as cancers, including renal cell carcinoma, where ALKBH5 and FTO protein and mRNA levels are downregulated in kidney tissues. This reduced expression correlates with poorer overall and tumor-specific survival following nephrectomy (63).

### 4.3 m6A readers: YTHDF and YTHDC families

YTHDF1 is highly expressed in RA synovial tissues, where its levels correlate positively with disease progression (64). It binds to m6A-modified transcripts of proinflammatory mediators (TNF-α and IL-6), increasing their translation and thus promoting synovial inflammation and hyperplasia (65). Moreover, YTHDF2 is also upregulated in RA synovium and promotes chondrocyte apoptosis and cartilage degradation by destabilizing anti-inflammatory factor mRNAs. YTHDF3 exacerbates pathogenic effects by facilitating both YTHDF1-mediated translation of proinflammatory mRNAs and YTHDF2-mediated degradation of protective transcripts (66). The nuclear-enriched readers YTHDC1 and YTHDC2 bridge nuclear RNA processing with cytoplasmic effector functions (**Figure 1**). In RA, YTHDC1 is primarily located in the nuclei of synovial and immune cells, where it binds pre-mRNAs of key NF-κB pathway components and promotes sustained NF-κB activation and proinflammatory gene expression (67).

## 5 Mechanism of metabolic reprogramming in RA

The metabolic landscape of RA is shaped by a dynamic ‘tug-of-war’ between m6A writers, erasers, and readers within a single synovial effector cell. The kinetics of m6A turnover on metabolic transcripts and the threshold modification levels required for functional consequences remain important open questions. In the nucleus, the competitive balance between METTL3-mediated methylation and FTO-mediated demethylation determines the methylation status of nascent transcripts. Once exported to the cytoplasm, these m6A-modified mRNAs are either translated by YTHDF1 to fuel metabolic flux or targeted for decay by YTHDF2. For instance, the net methylation level of HK2 mRNA directly couples this epitranscriptomic machinery to the glycolytic output of RA FLS. Notably, while the dysregulation of these m6A regulators is firmly established in human RA synovial biopsies (clinical evidence), the underlying molecular interactions have been primarily elucidated through CIA models and *in vitro* primary cultures (preclinical evidence). This coordinated network orchestrates the hallmarks of metabolic reprogramming discussed below.

### 5.1 Glucose metabolism reprogramming

**Figure 2:**
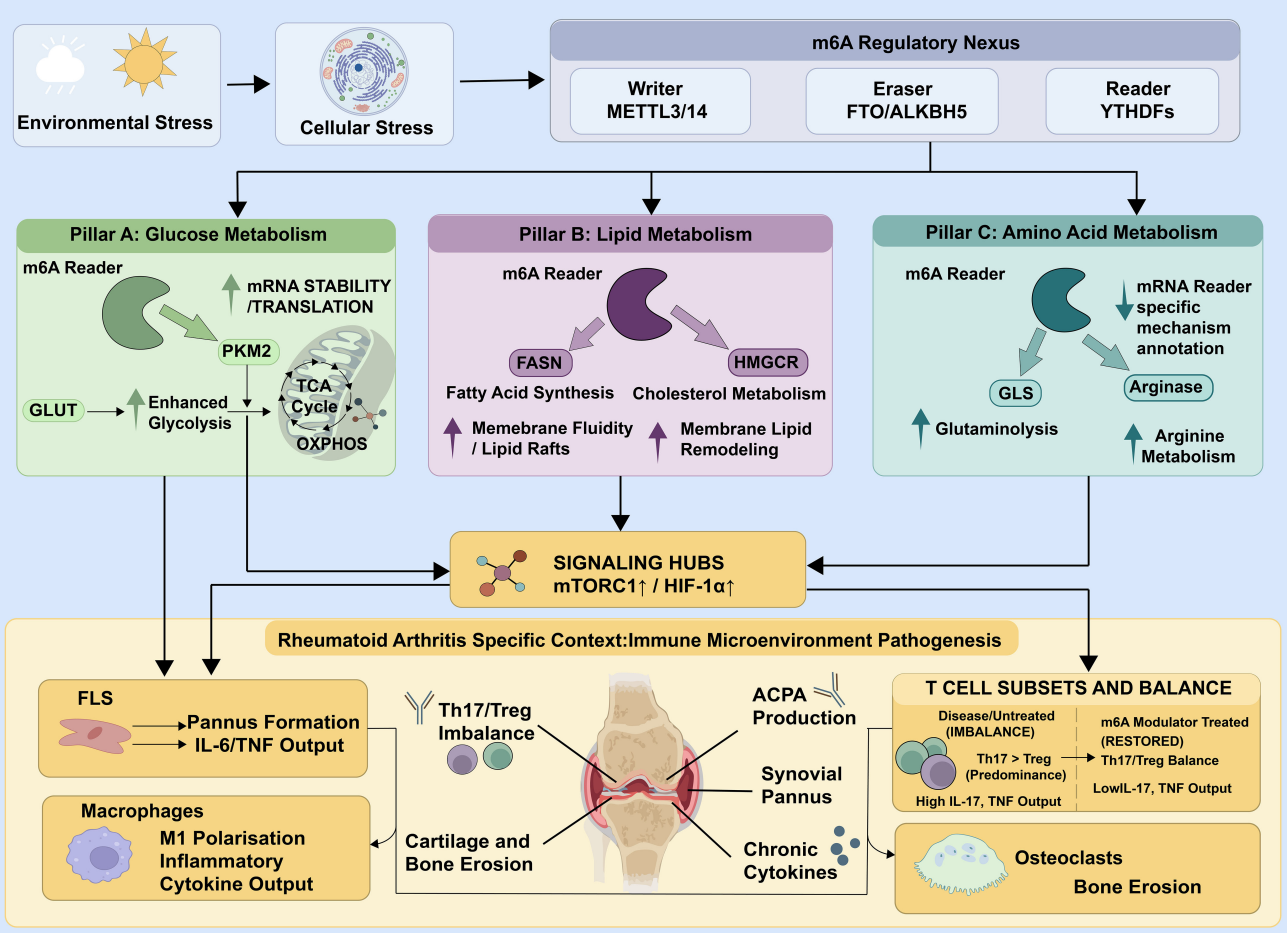
The m6A regulatory network links metabolic rewiring to immune−driven pathology in rheumatoid arthritis. Environmental and cellular stressors impinge on the core m6A machinery—writers (METTL3/14), erasers (FTO/ALKBH5), and readers (YTHDF proteins)—which in turn shapes the fate of transcripts involved in three metabolic pillars. In glucose metabolism, m6A−mediated control of PKM2 and GLUT expression bolsters glycolysis and TCA cycle flux. Lipid metabolism is fine−tuned through m6A−sensitive regulation of FASN and HMGCR, altering fatty acid synthesis, cholesterol handling, and membrane architecture. Amino acid pathways, including glutaminolysis and arginine utilization, are also under m6A influence. These metabolic shifts feed into signaling hubs centered on mTORC1 and HIF−1α, reinforcing a pro−inflammatory milieu within the rheumatoid joint. Downstream, the altered metabolism supports aggressive FLS behavior, pannus expansion, and sustained IL−6/TNF output. In parallel, it skews macrophages toward an M1−like phenotype, disrupts the Th17/Treg balance in T cells, and fuels osteoclast−mediated bone erosion, collectively driving the chronic destructive cycle of RA.

In RA pathogenesis, the reprogramming of glucose metabolism, specifically the increase in aerobic glycolysis, is considered the key metabolic phenotype driving synovial inflammation and tissue destruction (68). This phenomenon is commonly found in various important effector cells, adapting to their pathogenic activities via distinct signaling pathways, thus promoting RA progression (**Figure 2**). In adaptive immunity, the metabolic remodeling of activated T cells is essential for both initiating and maintaining the inflammatory cycle. Proinflammatory Th17 cell subsets significantly activate the PI3K/Akt/mTOR signaling pathway after T cell receptor activation, coordinating metabolic reprogramming to support rapid proliferation and effector function (69, 70). This metabolic transition upregulates core glycolytic components, such as glucose transporter 1 (GLUT1) and HK2, thus increasing lactate production from glucose carbon. The primary significance of this metabolic transition is the rapid production of ATP to support cellular functions, as well as providing essential biosynthetic precursors for Th17 cell clonal proliferation and substantial manufacture of its effector chemical IL-17 (71). This mechanistically couples metabolic flux to proinflammatory effector output. Prior to metabolic adaptation, fibroblast-like synoviocytes receive activating signals from infiltrating immune cells. Notably, age-associated B cells (ABCs) contribute to RA pathogenesis by secreting TNF-α, which activates FLS through ERK1/2 and JAK-STAT1 pathways, establishing a pro-inflammatory milieu that subsequently drives metabolic reprogramming (72). The metabolic adaptation of FLS simultaneously reacts to the distinct microenvironment of the joint cavity. Moreover, due to hypoxia and the accumulation of inflammatory factors resulting from synovial tissue proliferation, FLS predominantly depend on the stable expression of hypoxia-inducible factor HIF-1α to begin their metabolism (47). It has been confirmed that HIF-1α can directly bind to and activate the transcription of glycolytic genes such as PFKFB3 and LDHA. This provides FLS with a “Warburg effect” similar to that observed in tumor cells, which not only promotes their aberrant proliferation but also directly transforms the resultant glycolytic flux into the synthesis and secretion of matrix metalloproteinases (MMP-3, MMP-13), directly linking their metabolic state to invasive and cartilage-destructive potential (73). Further, cells sense their energy status *via* AMPK, promote growth signals through the MYC oncogene, and induce prolonged responses against inflammatory and stress signals in the microenvironment *via* epigenetic mechanisms, such as histone acetylation (74, 75). The coordinated activity of these complex regulatory networks transforms glucose metabolism from a mere energy source to a crucial control hub, which is now considered crucial for RA pathology.

### 5.2 Lipid metabolic reprogramming

In the synovial microenvironment of RA, lipid metabolic reprogramming represents an active adaptive process through which multiple cell types adjust their biosynthetic and catabolic pathways to meet pathogenic demands, rather than simply storing energy (76). By coordinating lipid synthesis, degradation, and transport, this adaptive shift sustains chronic inflammation and contributes to the progressive destruction of tissues. Dysregulated lipid metabolism in FLS plays a key role in this process. Elevated expression of FASN drives excessive *de novo* fatty acid synthesis (**Figure 2**). These newly formed lipids serve two essential roles: they provide structural substrates for membrane phospholipids to support abnormal FLS proliferation and persistent activation, and they are closely linked to the increased secretion of proinflammatory mediators such as TNF-α and IL-6 (77, 78). Furthermore, the *de novo* formation of fatty acids such as palmitate is required for the assembly and activity of key inflammatory signaling platforms, including the NLRP3 inflammasome (79). Thus, lipid synthesis not only meets the biosynthetic requirements of proliferating synoviocytes but also strengthens the inflammatory microenvironment.

This metabolic regulation extends beyond cell-intrinsic behavior and shapes intercellular signaling across the synovial niche (80). For example, microbial-derived short-chain fatty acids and dietary polyphenol metabolites can reshape immune cell activity and epigenetic states on a systemic level (81). Research from *in vitro* co-culture systems demonstrates that a CCR2 antagonist can shift macrophages toward an M2 phenotype by reducing the secretion of proinflammatory cytokines, underscoring the ability of metabolic signals to direct immune polarization (78). These findings highlight RA-FLS as metabolic and inflammatory hubs that transmit polarizing signals to neighboring immune cells through surface CCR2. Infiltrating immune cells undergo distinct forms of lipid metabolic remodeling to support their functional roles (**Figure 2**). M1 macrophages increase fatty acid β-oxidation to secure sufficient ATP for high-intensity inflammatory responses, while activated CD4^+^ T cells upregulate lipid transporters such as CD36 to increase uptake of extracellular lipids (82, 83). Furthermore, innate immune sensors such as galectin-3 recognize pathogen-associated molecular patterns and rapidly induce glycolysis through mTORC1 activation, providing additional metabolic fuel for inflammatory macrophages (84). These exogenous fatty acids not only support clonal proliferation but, depending on their composition, favor differentiation toward the Th17 proinflammatory lineage, highlighting inflammatory pathology. These coordinated lipid-dependent shifts are tightly regulated by transcription factors, including SREBP, and metabolic pathways, such as the mTOR pathway.

### 5.3 Amino acid metabolic reprogramming

In the metabolic reprogramming landscape of RA, amino acid metabolism, specifically the glutamine-related pathways, provides the indispensable metabolic foundation for the malignant proliferation and survival adaptation of pathological synovial cells (85). By modulating glutamine uptake and utilization, this pathway supplies essential biosynthetic precursors and confers resistance to oxidative stress, thus promoting the chronic progression of RA (86). From the perspective of FLS, these cells exhibit profound glutamine dependence (87). The key mechanism is the upregulation of the glutamine transporter SLC1A5 on the FLS membrane, which substantially increases glutamine uptake from the microenvironment to support rapid proliferation (**Figure 2**). Furthermore, intracellular glutamine is metabolized *via* two principal pathways: it acts as an anaplerotic substrate to TCA cycle intermediates, and it stimulates the mTOR signaling pathway by synergizing with other amino acid metabolic pathways, such as branched-chain amino acid metabolism (88). This activation directly promotes cellular proliferation and inflammatory responses, as well as integrates with increased glycolysis to form a coordinated “amino acid-glucose metabolic network” that collectively promotes RA progression.

Glutamine metabolism is crucial for cellular stress defense. The RA synovial microenvironment is characterized by substantial oxidative stress, with glutamine serving as the principal precursor for glutathione (GSH) production, the predominant intracellular antioxidant (89). The RA cells increase their GSH reserves by upregulating glutamine metabolic flux, improving their antioxidant capacity, and supporting survival under stress induced by the microenvironment and various treatments. This systemic metabolic adaptation is promoted by key transcription factors, including c-Myc and HIF-1α (90). It has been confirmed that pharmacological inhibition of glutamine metabolism (such as by glutaminase inhibitors) impairs RA-FLS’s proliferation and inflammatory cytokine secretion (**Figure 2**). Therefore, glutamine metabolic reprogramming is a crucial metabolic pathway in RA pathogenesis, and its inhibition is a promising therapeutic strategy with substantial translational potential.

## 6 Synergistic regulatory mechanisms of m6a methylation and metabolic reprogramming in RA

### 6.1 The m6A-metabolism axis in immune tolerance and autoimmunity

Beyond localized joint destruction, the breakdown of peripheral immune tolerance and subsequent autoantibody (e.g., ACPA, RF) production represent the inaugural events of RA. This systemic transition demands profound metabolic remodeling within lymphoid tissues. Specifically, m6A “writers” such as METTL3 dictate the metabolic fitness of dendritic cells (DCs) and T follicular helper (Tfh) cells (91, 92). By modifying transcripts of rate-limiting metabolic enzymes (93), m6A signaling licenses DC maturation and Tfh glycolytic shifts, thereby orchestrating aberrant germinal center responses. This energetically demanding microenvironment selectively supports autoreactive B cell survival, clonal expansion, and immunoglobulin class-switching. Consequently, the m6A-metabolism axis functions as a fundamental catalyst for early tolerance failure, preceding clinical synovitis (94) (**Table 3**).

**Table 3:** Integrated m6A-metabolic regulatory network linking epitranscriptomics to loss of tolerance and autoantibody production in RA.

m6A regulator	Specific enzyme	Target mRNA/pathway	RA cell type	Metabolic reprogramming	Functional outcome	Evidence
Writer	METTL3	HK2, GLUT1	FLS, Th17	Glycolysis	Promotes Proliferation & IL-17; drives autoreactivity	(51, 52)
Eraser	FTO	HIF-1α	FLS	Glycolysis	Upregulates PFK1/LDHA; supports autoAb niche	(27)
Writer	METTL14	FASN	FLS	Lipid Synthesis	Enhances FA synthesis; fuels Ag presentation	(53)
Eraser	ALKBH5	CPT1	Macrophages	Lipid β-oxidation	Sustains M1 polarization; breaks tolerance	(102)
Writer	METTL3	SLC1A5	FLS, T cells	Glutaminolysis	Increases Gln uptake; drives autoreactive T cells	(86, 101)
Reader	YTHDF2	GLS	Macrophages	Glutaminolysis	Decreases IL-1β; loss exacerbates Th17 autoimmunity	(107, 109)
Writer/Reader	METTL3/YTHDF1	Osteoclastogenic factors	Osteoclasts	Energy Metabolism	Drives bone resorption; fuels ACPA/RF	(112, 114, 115)

### 6.2 m6A methylation regulates glycolytic reprogramming

**Figure 3:**
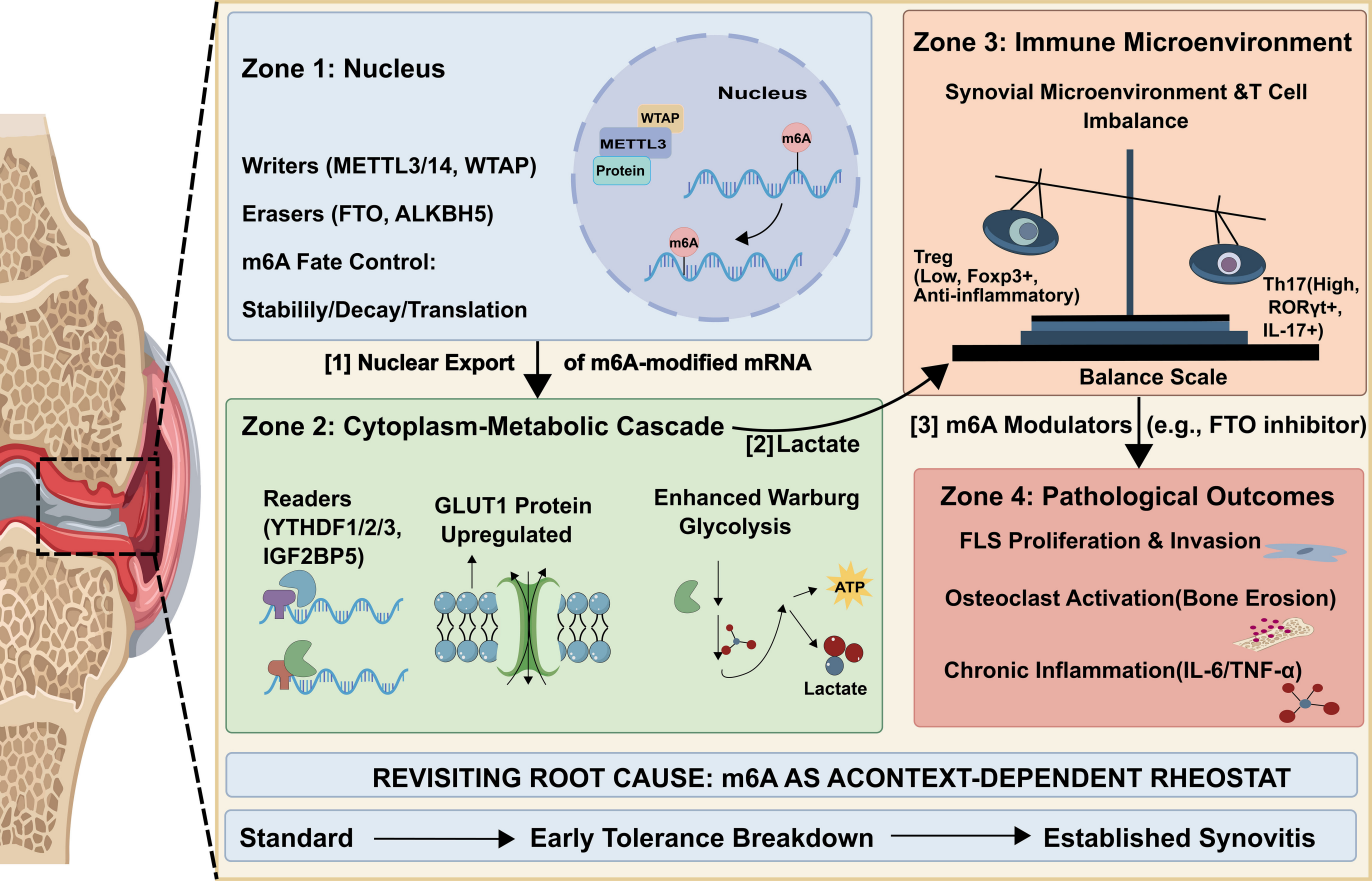
The m6A methylation-metabolic reprogramming axis in RA. Zone 1: m6A writers (METTL3/14, WTAP) and erasers (FTO, ALKBH5) control mRNA fate. [1] Nuclear export of m6A-modified mRNA. Zone 2: Readers (YTHDF1/2/3, IGF2BP5) enhance GLUT1 expression and glycolytic activity, producing ATP and lactate. [2] Lactate enters the extracellular space. Zone 3: Lactate promotes a Th17/Treg imbalance in the synovial microenvironment. [3] m6A modulators (e.g., FTO inhibitors) restore this balance. Zone 4: Pathological outcomes include FLS proliferation and invasion, osteoclast-mediated bone erosion, and chronic inflammation (IL-6/TNF-α). Bottom bar: m6A function shifts from standard homeostasis through early tolerance breakdown to established synovitis.

m6A methylation directly orchestrates the expression of essential glycolytic enzymes, augmenting aerobic glycolytic capacity in RA cells. This immediately provides sufficient energy to meet the proliferative requirements and inflammatory responses of synovial tissue in RA. Elevated METTL3 expression in RA-FLS cells alters m6A modifications on mRNAs that encode important glycolytic enzymes (95). YTHDF1 recognizes these modified mRNAs in the cytoplasm and recruits translation initiation factors to increase translation efficiency, thus elevating HK2 and GLUT1 protein expression (96). Studies have revealed that METTL3 knockdown in RA-FLS significantly decreased HK2 mRNA m6A modification, reducing glycolytic rates. This substantially inhibits FLS proliferation, MMP-13 secretion, and articular cartilage erosion (**Figure 3**). Moreover, other than METTL3, FTO also crucially modulates glycolytic reprogramming (97). In RA synovial cells, FTO erases m6A marks on hypoxia-inducible factor HIF-1α mRNA, thereby blocking YTHDF2-mediated recognition and degradation and consequently stabilizing the transcript (98). This preservation of mRNA stability sustains HIF-1α protein expression, which subsequently upregulates glycolytic enzymes such as PFK1 and LDH (99). LDH facilitates the aberrant proliferation of RA-FLS in the inflammatory microenvironment. A testable hypothesis emerging from these findings is that the METTL3-HK2 axis operates as a feed-forward loop, wherein inflammatory cytokines upregulate METTL3 to enhance HK2 translation and lactate production, and lactate in turn amplifies METTL3 expression via histone lactylation. Therefore, FTO inhibition can accelerate HIF-1α mRNA degradation, disrupt glucose metabolism reprogramming, and decrease synovial inflammation (**Table 3**).

### 6.3 m6A-mediated control of lipid homeostasis and inflammatory signaling

m6A methylation allows RA cells to meet the requirement for “membrane structural raw materials-inflammatory mediator precursors” by modulating the RNA metabolism of lipid synthesis- and degradation-related genes (27, 100). In lipid synthesis, YTHDF3 recognizes METTL14-modified FASN mRNA, improving its translation efficiency (101). FASN is a key enzyme in fatty acid synthesis, and its increased expression promotes the synthesis of fatty acids, such as palmitic acid, thus providing phospholipid precursors for rapid FLS proliferation (**Table 3**). Evidence from cancer metabolism studies supports the role of m6A writers, such as METTL14, in the upregulation of lipid metabolism genes like FASN. ALKBH5 has a crucial role in lipid catabolism (**Figure 3**). In M1 macrophages, ALKBH5removes m6A modifications from carnitine palmitoyltransferase-1 (CPT1) mRNA (102). CPT1 is the rate-limiting enzyme in fatty acid β-oxidation. YTHDF2-mediated degradation of CPT1 mRNA is inhibited by ALKBH5 demethylation, thus sustaining CPT1 activity (103). This promotes macrophage energy production through intracellular lipids β-oxidation, while generating arachidonic acid. Moreover, ALKBH5 inhibition accelerates CPT1 degradation, resulting in an insufficient energy supply in M1 macrophages and reduced inflammatory activity.

### 6.4 Cell-specific tuning of amino acid flux

In RA, m6A methylation precisely governs glutamine metabolism through a cell-type-specific dual mechanism. The METTL3-YTHDF1 axis in FLS and activated T cells enhances the translation of m6A-modified SLC1A5 mRNA, thereby elevating glutamine uptake (104). Once imported, glutamine is converted by GLS into α-KG to fuel the TCA cycle via anaplerosis, providing carbon and nitrogen precursors for biomass synthesis and glutathione-mediated antioxidant defense (105, 106). In contrast, a feedback mechanism exists in RA macrophages where YTHDF2 recognizes m6A-modified GLS mRNA to promote its degradation, maintaining metabolic equilibrium (107) (**Table 3**). However, clinical data indicate that PBMCs from RA patients exhibit reduced YTHDF2 activity, leading to increased GLS stability and excessive glutamine degradation. This metabolic dysregulation is linked to ammonia accumulation and exacerbated IL-1β secretion, highlighting the association between m6A-driven amino acid flux and the key NLRP3 inflammatory axis (108–110).

### 6.5 Epitranscriptomic regulation of osteoclastogenesis and bone bioenergetics

m6A methylation significantly modulates articular bone destruction by exerting precise control over the differentiation and bioenergetic capacity of osteoclasts (52). A primary pro-resorptive pathway involves the METTL3-YTHDF1 axis, which enhances the translation of osteoclastogenic factor mRNAs to drive bone resorption (111–113) (**Table 3**). Given its mechanistic importance, this axis has emerged as a viable target for natural compounds aimed at protecting bone integrity. Conversely, METTL3 also activates a YTHDF2-dependent inhibitory pathway. Deletion of METTL3 increases the stability of Atp6v0d2 mRNA via YTHDF2, resulting in the formation of multinucleated yet functionally impaired osteoclasts with limited resorbing capacity (114). Furthermore, under inflammatory stress, METTL3 can impair osteoclast function by inducing mitochondrial dysfunction through the iNOS/NO signaling pathway (115). These findings reveal a sophisticated regulatory network where METTL3 exerts dual effects, balancing resorptive function against inflammation-induced metabolic stress (116).

### 6.6 Synthesis: m6A as a context-dependent rheostat

The m6A regulatory landscape in RA is defined by distinct, cell-type-specific signatures that dictate divergent pathologic outputs (**Table 3**). In FLS, a METTL3/FTO-mediated switch drives invasive phenotypes through metabolic adaptation (52, 78, 117). In contrast, m6A mechanisms in macrophages and T cells focus on inflammatory polarization and lineage decisions (55, 118). Within the synovial niche, ALKBH5 acts as a metabolic rheostat, suppressing NLRP3 activation to mitigate localized inflammation. Rather than following a universal template, m6A signaling functions as a context-dependent regulator, modulating its output to align with the unique metabolic pressures of the RA microenvironment.

Despite this cell-type specificity, several overarching principles emerge. First, the METTL3-YTHDF1 axis functions as a conserved pro-inflammatory module across FLS, Th17 cells, and M1 macrophages, consistently coupling m6A installation to enhanced translation of glycolytic and pro-inflammatory transcripts. Second, the master regulators HIF-1α and mTORC1 serve as central integrators. They receive inputs from both m6A-modified metabolic enzymes and inflammatory signals, and in turn amplify m6A writer expression. This establishes self-reinforcing feed-forward loops that sustain pathogenic reprogramming. Third, ALKBH5 appears to act as a context-dependent ‘brake’ on excessive inflammation. Its protective role in chondrocytes and M1 macrophages contrasts with the pro-inflammatory dominance of METTL3, suggesting that therapeutic restoration of m6A homeostasis may require simultaneous modulation of multiple regulators rather than single-node inhibition. These principles provide a conceptual scaffold for understanding how epitranscriptomic and metabolic dysregulation are coordinated across the heterogeneous cellular landscape of the RA joint.

## 7 m6A Methylation and metabolic reprogramming targets for RA therapy

### 7.1 Therapeutic modulation of the m6a landscape

Emerging evidence suggests that conventional synthetic DMARDs (csDMARDs) may exert their therapeutic effects by indirectly modulating the m6A epitranscriptome. Methotrexate (MTX), the anchor therapy for RA, functions as a potent folate antagonist, thereby disrupting one-carbon metabolism and the methionine cycle (119). Given that S-adenosylmethionine (SAM) serves as the universal methyl donor for m6A writers (120), MTX treatment potentially constrains the global m6A methylome by limiting SAM availability. This “metabolic hijacking” could counteract the pathologically elevated METTL3 activity observed in RA synovial tissues (52). Furthermore, glucocorticoids and certain targeted synthetic DMARDs have been implicated in restoring m6A homeostasis by rebalancing the expression of erasers like FTO (121). Similarly, biologic DMARDs such as TNF-α and IL-6 inhibitors may indirectly normalize m6A patterns by alleviating the inflammatory and hypoxic drivers of metabolic reprogramming (122).

Beyond these indirect effects, the development of direct small-molecule m6A inhibitors (e.g., METTL3 inhibitor STM2457, FTO inhibitor FB23-2) offers a promising avenue for “epitranscriptomic corrective therapy.” However, as summarized in **Table 4**, all such direct m6A modulators remain at the preclinical stage in RA models, with no clinical trials yet reported. Bridging this translational gap represents a key priority for the field.

**Table 4:** Current research status of m6a modulators in rheumatoid arthritis.

Modulator	Intervention	Mechanism	Model/system	Status	Outcome	Ref
METTL3	STM2457	Inhibitor	CIA mice, RA-FLS	Preclinical	Reduces inflammation and glycolysis	(51, 52)
METTL3	siRNA	Knockdown	RA-FLS, CIA mice	Preclinical	Decreases HK2 and MMPs	(52)
FTO	FB23-2	Inhibitor	RA-FLS, CIA mice	Preclinical	Reduces synovitis and erosion	(27)
FTO	Natural compounds	Inhibitor	Macrophages, FLS	Preclinical	Decreases cytokines	(57)
ALKBH5	Overexpression	Activation	Chondrocytes, CIA	Preclinical	Increases cartilage protection	(59, 60)
YTHDF1	siRNA	Knockdown	RA-FLS, T cells	Preclinical	Decreases TNF-α and IL-6	(64)
YTHDF2	Genetic modulation	Stabilization	PBMCs, CIA mice	Preclinical	Loss increases IL-1β	(105)
IGF2BP2	CWI1-2	Inhibitor	Cancer models	Preclinical (cancer)	Regulates glutamine metabolism	(31)
MTX	Folate antagonist	SAM depletion	RA patients	Approved	Indirect m6A effect	(119)
JAKi	Tofacitinib/Baricitinib	JAK-STAT blockade	RA patients	Approved	Restores metabolic balance	(122)

### 7.2 Deepening intervention strategies and mechanisms at key targets

Several studies have identified different core m6A-metabolism regulatory axes as priority therapeutic targets, where the METTL3-HK2 axis indicates significant potential (123). In RA-FLS, METTL3 inhibition reduces m6A modification of proinflammatory and proliferative genes, as well as specifically decreases the translation efficiency of HK2, a key glycolytic enzyme (52). This effectively terminates the signaling switch that governs malignant cellular activity. In the hypoxia-inflammation microenvironment of RA, the inhibition of FTO activity enhances the identification and degradation of HIF-1α mRNA by YTHDF2 (124). This disturbs the expression equilibrium of downstream glycolytic genes, including PFK1 and LDHA, fundamentally compromising the metabolic capacity of diseased cells to adapt to unfavorable microenvironments. Moreover, targeting the ALKBH5-CPT1 axis in macrophages enhances fatty acid β-oxidation by stabilizing CPT1 mRNA, thus modulating energy metabolism and inflammatory phenotypes (102, 125). This approach expands the scope of targeted therapy beyond carbohydrate metabolism.

### 7.3 Synergistic intervention: from single-point targeting to network regulation

Due to the complexity of m6A and metabolic networks, combination interventions targeting synergistic points improve therapeutic efficacy while overcoming compensatory mechanisms (126). For example, dual inhibition of glycolysis and glutaminolysis disrupts metabolic flux in RA cells (127). Furthermore, co-targeting epitranscriptomic regulators (e.g., FTO) alongside hypoxia response pathways represents a promising strategy to compromise cellular metabolism (128), though specific FTO-HIF-1α synergistic mechanisms in RA require further validation.

### 7.4 Clinical translation prospects and challenges

The translation of the aforementioned targets from theory to clinical practice is both challenging and beneficial. A proof-of-concept study has validated the therapeutic efficiency of targeting metabolic reprogramming in RA (129). This animal model study showed that a targeted delivery system carrying roburic acid could effectively reprogram proinflammatory macrophages in the joint, altering their metabolism from glycolysis to oxidative phosphorylation, promoting a shift from the M1 to the M2 phenotype, which alleviates RA symptoms. This success not only confirms metabolic reprogramming as a viable therapeutic target but also underscores the essential significance of targeted delivery techniques. Exosomes, due to their biocompatibility, low immunogenicity, and targeting properties, present a viable approach for the delivery of therapeutic compounds to specific joint cells. This possibility is demonstrated by exosomal non-coding RNAs (ncRNAs), which have significant therapeutic potential for RA and other bone metabolic disorders (130). Engineered exosomes offer a platform for co-delivering m6A modulators and metabolic inhibitors to the inflamed synovium, achieving synergistic targeting of the “epigenetic-metabolic” axis (131, 132). This strategy may complement existing therapies, particularly in refractory RA. Furthermore, this approach is supported by key m6A inhibitors from oncology research (e.g., METTL3 inhibitor STM2457 and FTO inhibitor FB23-2), providing valuable lead compounds for RA drug development (133). However, substantial obstacles remain beyond delivery efficiency. Systemic m6A modulation risks off-target effects on normal hematopoiesis and neural function, while achieving cell-type-specific targeting exceeds the precision of current inhibitors. An ideal strategy would selectively inhibit METTL3 in pathogenic FLS without disrupting ALKBH5 in chondrocytes, yet this remains technically challenging. Metabolic plasticity also raises the prospect of resistance. If glycolysis is blocked, cells may switch to fatty acid oxidation, necessitating combination strategies. The main challenge is to improve the efficiency of targeted drug delivery to diseased joint tissues, optimizing therapeutic effectiveness and reducing systemic side effects (134). This may require technology such as nanocarriers or intra-articular delivery (135). Moreover, precisely evaluating the unique impacts of these pharmaceuticals on various cellular subpopulations within the RA environment, including fibroblast-like synoviocytes, T cells, and macrophages, is an essential prerequisite for ensuring therapeutic safety and efficacy (136).

Beyond delivery challenges, successful clinical translation will require robust biomarkers for patient stratification and therapeutic monitoring. Currently, no validated biomarkers exist to identify which RA patients are most likely to benefit from m6A-or metabolism-targeted therapies. Potential candidates include circulating m6A-modified transcripts (e.g., cell-free HK2 or SLC1A5 mRNA), exosomal m6A regulators (e.g., METTL3 or FTO protein levels in synovial fluid-derived exosomes), or non-invasive imaging of synovial glycolytic flux via FDG-PET. Longitudinal studies correlating these candidate biomarkers with disease activity scores and treatment responses are urgently needed to enable precision medicine approaches within the m6A-metabolism-immunity axis. Looking forward, synthetic biology circuits that restore m6A homeostasis only within inflamed joints and artificial intelligence-driven network analysis may unlock novel therapeutic vulnerabilities, advancing precision epitranscriptomic therapy in RA.

## 8 Conclusion and limitations

This review establishes that the interaction between m6A methylation and metabolic reprogramming constitutes a central pathogenic axis in RA. The m6A regulatory network orchestrates the fate of transcripts involved in immune activation and synovial pathology, while metabolic adaptations provide the necessary energy and biosynthetic precursors. Notably, m6A modifications directly shape these metabolic pathways by modulating the expression and activity of key enzymes and transporters governing glucose, lipid, and amino acid metabolism.

This review asked whether m6A methylation constitutes a root cause of metabolic reprogramming in RA. The evidence synthesized herein suggests a nuanced answer. m6A appears neither as a universal initiator nor a passive bystander, but rather as a context-dependent rheostat that amplifies and sustains pathogenic metabolic states once autoimmunity is established. In early tolerance breakdown, m6A-driven metabolic remodeling licenses aberrant germinal center responses and autoantibody production. In established synovitis, m6A-mediated feed-forward loops perpetuate glycolytic flux and inflammatory effector functions. This perspective reframes m6A as a critical node through which genetic susceptibility, environmental stress, and metabolic demand converge to drive disease chronicity. Several limitations constrain the interpretation of this model. The synergistic relationship between m6A and metabolism remains incompletely elucidated, particularly regarding reader-specific regulatory patterns and nodal control points. The field lacks tools for real-time surveillance of these interactions during disease progression. Methodological constraints further complicate existing data: most findings derive from normoxic *in vitro* systems diverging from the hypoxic RA synovium, and publication bias likely obscures negative results. Importantly, key mechanisms have primarily been elucidated in oncology or general inflammation models, and their precise translatability to the cytokine-driven autoimmune environment of RA joints requires rigorous validation. Before clinical translation, confirmatory studies must be performed using patient-derived tissues, primary synovial fibroblast cultures, and relevant animal models such as collagen-induced arthritis. To definitively test causality, future studies should prioritize three key questions: whether temporally controlled, cell-type-specific deletion of METTL3 before disease onset can prevent metabolic reprogramming and arthritis development in CIA models; how genome-wide m6A occupancy on metabolic transcripts evolves during the transition from pre-RA to established synovitis in human longitudinal cohorts; and whether therapeutic restoration of m6A homeostasis can reverse established metabolic dysfunction and joint destruction in refractory RA.

## References

[1] Smolen JS Aletaha D Barton A Burmester GR Emery P Firestein GS . Rheumatoid arthritis. Nat Rev Dis Primers. (2018) 4:18001. doi: 10.1038/nrdp.2018.1. PMID: 29417936

[2] Malmström V Catrina AI Klareskog L . The immunopathogenesis of seropositive rheumatoid arthritis: from triggering to targeting. Nat Rev Immunol. (2017) 17:60–75. doi: 10.1038/nri.2016.124. PMID: 27916980

[3] Krabben A Huizinga TW Mil AH . Biomarkers for radiographic progression in rheumatoid arthritis. Curr Pharm Des. (2015) 21:147–69. doi: 10.2174/1381612820666140825122525. PMID: 25163742

[4] Haville S Deane KD . Pre-RA: Can early diagnosis lead to prevention? Best Pract Res Clin Rheumatol. (2022) 36:101737. doi: 10.1016/j.berh.2021.101737. PMID: 34991984 PMC8977282

[5] Akram M Daniyal M Sultana S Owais A Akhtar N Zahid R . Traditional and modern management strategies for rheumatoid arthritis. Clin Chim Acta. (2021) 512:142–55. doi: 10.1016/j.cca.2020.11.003. PMID: 33186593

[6] Conran C Kolfenbach J Kuhn K Striebich C Moreland L . A review of difficult-to-treat rheumatoid arthritis: definition, clinical presentation, and management. Curr Rheumatol Rep. (2023) 25:285–94. doi: 10.1007/s11926-023-01117-6. PMID: 37776482

[7] Chi XK Xu XL Chen BY Su J Du YZ . Combining nanotechnology with monoclonal antibody drugs for rheumatoid arthritis treatments. J Nanobiotechnology. (2023) 21:105. doi: 10.1186/s12951-023-01857-8. PMID: 36964609 PMC10039584

[8] Scherer HU Häupl T Burmester GR . The etiology of rheumatoid arthritis. J Autoimmun. (2020) 110:102400. doi: 10.1016/j.jaut.2019.102400. PMID: 31980337

[9] Huang Y Xue Q Chang J Wang Y Cheng C Xu S . M6A methylation modification in autoimmune diseases, a promising treatment strategy based on epigenetics. Arthritis Res Ther. (2023) 25:189. doi: 10.1186/s13075-023-03149-w. PMID: 37784134 PMC10544321

[10] Jiang X Liu B Nie Z Duan L Xiong Q Jin Z . The role of m6A modification in the biological functions and diseases. Signal Transduce Target Ther. (2021) 6:74. doi: 10.1038/s41392-020-00450-x. PMID: 33611339 PMC7897327

[11] Kondo N Kuroda T Kobayashi D . Cytokine networks in the pathogenesis of rheumatoid arthritis. Int J Mol Sci. (2021) 22:10922. doi: 10.3390/ijms222010922. PMID: 34681582 PMC8539723

[12] Flamand MN Tegowski M Meyer KD . The proteins of mRNA modification: writers, readers, and erasers. Annu Rev Biochem. (2023) 92:145–73. doi: 10.1146/annurev-biochem-052521-035330. PMID: 37068770 PMC10443600

[13] Xu L Shen T Li Y Wu X . The role of M6A modification in autoimmunity: emerging mechanisms and therapeutic implications. Clin Rev Allergy Immunol. (2025) 68:29. doi: 10.1007/s12016-025-09041-6. PMID: 40085180

[14] Abu-Tawil HI Picavet LW van Vroonhoven ECN Bodelón A Scholman RC Ter Haar N . Reduced expression of m6A demethylases FTO and ALKBH5 in monocytes from the site of inflammation in patients with juvenile idiopathic arthritis. Int J Mol Sci. (2025) 26:9248. doi: 10.3390/ijms26189248. PMID: 41009809 PMC12471030

[15] Wu G Yang C Huang Y Liu B Zhai H Cao Z . Histone lactylation promotes rheumatoid arthritis progression by increasing NFATc2 expression and the production of anti-lactylated histone autoantibodies. Nat Commun. (2025) 16:9034. doi: 10.1038/s41467-025-64096-5. PMID: 41073397 PMC12514170

[16] Zhu L Zhu X Wu Y . Effects of glucose metabolism, lipid metabolism, and glutamine metabolism on tumor microenvironment and clinical implications. Biomolecules. (2022) 12:580. doi: 10.3390/biom12040580. PMID: 35454171 PMC9028125

[17] An Y Duan H . The role of m6A RNA methylation in cancer metabolism. Mol Cancer. (2022) 21:14. doi: 10.1186/s12943-022-01500-4. PMID: 35022030 PMC8753874

[18] Shi H Wei J He C . Where, when, and how: context-dependent functions of RNA methylation writers, readers, and erasers. Mol Cell. (2019) 74:640–50. doi: 10.1016/j.molcel.2019.04.025. PMID: 31100245 PMC6527355

[19] Sendinc E Shi Y . RNA m6A methylation across the transcriptome. Mol Cell. (2023) 83:428–41. doi: 10.1016/j.molcel.2023.01.006. PMID: 36736310

[20] Alarcón CR Lee H Goodarzi H Halberg N Tavazoie SF . N6-methyladenosine marks primary microRNAs for processing. Nature. (2015) 519:482–5. doi: 10.1038/nature14281. PMID: 25799998 PMC4475635

[21] Oerum S Meynier V Catala M Tisné C . A comprehensive review of m6A/m6Am RNA methyltransferase structures. Nucleic Acids Res. (2021) 49:7239–55. doi: 10.1093/nar/gkab378. PMID: 34023900 PMC8287941

[22] Zeng C Huang W Li Y Weng H . Roles of METTL3 in cancer: mechanisms and therapeutic targeting. J Hematol Oncol. (2020) 13:117. doi: 10.1186/s13045-020-00951-w. PMID: 32854717 PMC7457244

[23] Zhang D Xu T Gao X Qu Y Su X . Methyltransferase-like 3-mediated RNA N6-methyladenosine contributes to immune dysregulation: diagnostic biomarker and therapeutic target. Front Immunol. (2025) 16:1523503. doi: 10.3389/fimmu.2025.1523503. PMID: 40196133 PMC11973086

[24] Fan Y Li X Sun H Gao Z Zhu Z Yuan K . Role of WTAP in cancer: from mechanisms to the therapeutic potential. Biomolecules. (2022) 12:1224. doi: 10.3390/biom12091224. PMID: 36139062 PMC9496264

[25] Zhu S Li Z . VIRMA-mediated the m6A methylation of SCD facilitates Wilms' tumor progression via AMPK pathway. DNA Cell Biol. (2025) 44:229–37. doi: 10.1089/dna.2024.0288. PMID: 40040483

[26] Murakami S Jaffrey SR . Hidden codes in mRNA: control of gene expression by m6A. Mol Cell. (2022) 82:2236–51. doi: 10.1016/j.molcel.2022.05.029. PMID: 35714585 PMC9216239

[27] Li R Kuang Y Niu Y Zhang S Chen S Su F . FTO-mediated RNA m6A methylation regulates synovial aggression and inflammation in rheumatoid arthritis. Biochim Et Biophys Acta Biochim Biophys Acta Mol Basis Dis. (2024) 1870:167341. doi: 10.1016/j.bbadis.2024.167341. PMID: 39025373

[28] Hatipoglu OF Hirohata S Yaykasli KO Cilek MZ Demircan K Shinohata R . The 3'-untranslated region of ADAMTS1 regulates its mRNA stability. Acta Med Okayama. (2009) 63:79–85. doi: 10.18926/AMO/31831. PMID: 19404339

[29] Wu R Jiang D Wang Y Wang X . N (6)-methyladenosine (m(6)A) methylation in mRNA with a dynamic and reversible epigenetic modification. Mol Biotechnol. (2016) 58:450–9. doi: 10.1007/s12033-016-9947-9. PMID: 27179969

[30] Phani NM Vohra M Rajesh S Adhikari P Nagri SK D'Souza SC . Implications of critical PPARγ2, ADIPOQ and FTO gene polymorphisms in type 2 diabetes and obesity-mediated susceptibility to type 2 diabetes in an Indian population. Mol Genet Genomics. (2016) 291:193–204. doi: 10.1007/s00438-015-1097-4. PMID: 26243686

[31] Weng H Huang F Yu Z Chen Z Prince E Kang Y . The m6A reader IGF2BP2 regulates glutamine metabolism and represents a therapeutic target in acute myeloid leukemia. Cancer Cell. (2022) 40:1566–1582.e10. doi: 10.1016/j.ccell.2022.10.004. PMID: 36306790 PMC9772162

[32] Zaccara S Jaffrey SR . A unified model for the function of YTHDF proteins in regulating m6A-modified mRNA. Cell. (2020) 181:1582–1595.e18. doi: 10.1016/j.cell.2020.05.012. PMID: 32492408 PMC7508256

[33] Wang X Zhao BS Roundtree IA Lu Z Han D Ma H . N (6)-methyladenosine modulates messenger RNA translation efficiency. Cell. (2015) 161:1388–99. doi: 10.1016/j.cell.2015.05.014. PMID: 26046440 PMC4825696

[34] Shi H Wang X Lu Z Zhao BS Ma H Hsu PJ . YTHDF3 facilitates translation and decay of N6-methyladenosine-modified RNA. Cell Res. (2017) 27:315–28. doi: 10.1038/cr.2017.15. PMID: 28106072 PMC5339834

[35] Xiao W Adhikari S Dahal U Chen YS Hao YJ Sun BF . Nuclear m(6)a reader YTHDC1 regulates mRNA splicing. Mol Cell. (2016) 61:507–19. doi: 10.1016/j.molcel.2016.01.012. PMID: 26876937

[36] Freemerman AJ Johnson AR Sacks GN Milner JJ Kirk EL Troester MA . Metabolic reprogramming of macrophages: glucose transporter 1 (GLUT1)-mediated glucose metabolism drives a proinflammatory phenotype. J Biol Chem. (2014) 289:7884–96. doi: 10.1074/jbc.M113.522037. PMID: 24492615 PMC3953299

[37] Vaupel P Schmidberger H Mayer A . The Warburg effect: essential part of metabolic reprogramming and central contributor to cancer progression. Int J Radiat Biol. (2019) 95:912–9. doi: 10.1080/09553002.2019.1589653. PMID: 30822194

[38] Cao Y Rathmell JC Macintyre AN . Metabolic reprogramming towards aerobic glycolysis correlates with greater proliferative ability and resistance to metabolic inhibition in CD8 versus CD4 T cells. PloS One. (2014) 9:e104104. doi: 10.1371/journal.pone.0104104. PMID: 25090630 PMC4121309

[39] Faubert B Boily G Izreig S Griss T Samborska B Dong Z . AMPK is a negative regulator of the warburg effect and suppresses tumor growth *in vivo*. Cell Metab. (2013) 17:113–24. doi: 10.1016/j.cmet.2012.12.001. PMID: 23274086 PMC3545102

[40] Edmunds LR Sharma L Wang H Kang A d'souza S Lu J . c-myc and AMPK control cellular energy levels by cooperatively regulating mitochondrial structure and function. PloS One. (2015) 10:e0134049. doi: 10.1371/journal.pone.0134049. PMID: 26230505 PMC4521957

[41] Ito A . Lipid metabolic reprogramming in immune regulation and chronic inflammatory diseases. Endocr J. (2025) 72:979–85. doi: 10.1507/endocrj.EJ25-0180 PMC12436075 40436800

[42] Ringel AE Drijvers JM Baker GJ Catozzi A García-Cañaveras JC Gassaway BM . Obesity shapes metabolism in the tumor microenvironment to suppress anti-tumor immunity. Cell. (2020) 183:1848–1874.e26. doi: 10.1016/j.cell.2020.11.009. PMID: 33301708 PMC8064125

[43] Han Z Shen Y Yan Y Bin P Zhang M Gan Z . Metabolic reprogramming shapes post-translational modification in macrophages. Mol Aspects Med. (2025) 102:101338. doi: 10.1016/j.mam.2025.101338. PMID: 39977975

[44] Tan SK Hougen HY Merchan JR Gonzalgo ML Welford SM . Fatty acid metabolism reprogramming in ccRCC: mechanisms and potential targets. Nat Rev Urol. (2023) 20:48–60. doi: 10.1038/s41585-022-00654-6. PMID: 36192502 PMC10826284

[45] Zhang Q Wei T Jin W Yan L Shi L Zhu S . Deficiency in SLC25A15, a hypoxia-responsive gene, promotes hepatocellular carcinoma by reprogramming glutamine metabolism. J Hepatol. (2024) 80:293–308. doi: 10.1016/j.jhep.2023.10.024. PMID: 38450598

[46] Garcia-Carbonell R Divakaruni AS Lodi A Vicente-Suarez I Saha A Cheroutre H . Critical role of glucose metabolism in rheumatoid arthritis fibroblast-like synoviocytes. Arthritis Rheumatol. (2016) 68:1614–26. doi: 10.1002/art.39608. PMID: 26815411 PMC4963240

[47] Wang Z Wu X Chen HN Wang K . Amino acid metabolic reprogramming in tumor metastatic colonization. Front Oncol. (2023) 13:1123192. doi: 10.3389/fonc.2023.1123192. PMID: 36998464 PMC10043324

[48] Yoo HC Yu YC Sung Y Han JM . Glutamine reliance in cell metabolism. Exp Mol Med. (2020) 52:1496–516. doi: 10.1038/s12276-020-00504-8. PMID: 32943735 PMC8080614

[49] Wan L Liu J Huang C Zhu Z Wang K Sun G . Comprehensive analysis and functional characteristics of differential expression of N6-methyladenosine methylation modification in the whole transcriptome of rheumatoid arthritis. Mediators Inflammation. (2022) 2022:4766992. doi: 10.1155/2022/4766992. PMID: 36330380 PMC9626244

[50] Miao T Qiu Y Chen J Li P Li H Zhou W . METTL3 knockdown suppresses RA-FLS activation through m6A-YTHDC2-mediated regulation of AMIGO2. Biochim Biophys Acta Mol Basis Dis. (2024) 1870:167112. doi: 10.1016/j.bbadis.2024.167112 38432455

[51] Shi W Zheng Y Luo S Li X Zhang Y Meng X . METTL3 promotes activation and inflammation of FLSs through the NF-κB signaling pathway in rheumatoid arthritis. Front Med (Lausanne). (2021) 8:607585. doi: 10.3389/fmed.2021.607585. PMID: 34295905 PMC8290917

[52] Su Y Wu Z Liu Y Liu X Kang J Jia J . Increased m6A RNA methylation and METTL3 expression may contribute to the synovitis progression of rheumatoid arthritis. Exp Cell Res. (2024) 442:114237. doi: 10.1016/j.yexcr.2024.114237. PMID: 39245197

[53] Li X Xu X Zhang Q Ling M Li X Tan X . METTL14 promotes fibroblast-like synoviocytes activation via the LASP1/SRC/AKT axis in rheumatoid arthritis. Am J Physiol Cell Physiol. (2023) 324:C1089–C100. doi: 10.1152/ajpcell.00575.2022. PMID: 36878846

[54] Yang H Li Y Huang L Fang M Xu S . The epigenetic regulation of RNA N6-methyladenosine methylation in glycolipid metabolism. Biomolecules. (2023) 13:273. doi: 10.3390/biom13020273 36830642 PMC9953413

[55] Jiang Q Wang X Wu Y Cui D . The m(6)A modification in T helper cells regulates the pathogenesis of autoimmune diseases. J Inflammation Res. (2025) 18:13159–72. doi: 10.2147/jir.s537243. PMID: 40994654 PMC12456315

[56] Zhu W Wang JZ Wei JF Lu C . Role of m6A methyltransferase component VIRMA in multiple human cancers (review). Cancer Cell Int. (2021) 21:172. doi: 10.1186/s12935-021-01868-1. PMID: 33731118 PMC7968318

[57] Luo Q Gao Y Zhang L Rao J Guo Y Huang Z . Decreased ALKBH5, FTO, and YTHDF2 in peripheral blood are as risk factors for rheumatoid arthritis. BioMed Res Int. (2020) 2020:5735279. doi: 10.1155/2020/5735279. PMID: 32884942 PMC7455827

[58] Li G Fang Y Xu N Ding Y Liu D . Fibroblast-like synoviocytes-derived exosomal circFTO deteriorates rheumatoid arthritis by enhancing N6-methyladenosine modification of SOX9 in chondrocytes. Arthritis Res Ther. (2024) 26:66. doi: 10.1186/s13075-024-03290-0. PMID: 38388473 PMC10882813

[59] Zheng H AiHaiti Y Cai Y Yuan Q Yang M Li Z . The m6A/m1A/m5C-related methylation modification patterns and immune landscapes in rheumatoid arthritis and osteoarthritis revealed by microarray and single-cell transcriptome. J Inflammation Res. (2023) 16:5001–25. doi: 10.2147/JIR.S431076. PMID: 37933335 PMC10625757

[60] Xiao J Cai X Wang R Zhou W Ye Z . ALKBH5-YTHDF2 m6A modification axis inhibits rheumatoid arthritis progression by suppressing NLRP3. Biochem Biophys Res Commun. (2023) 668:70–6. doi: 10.1016/j.bbrc.2023.05.087. PMID: 37244037

[61] Wang K Liu F Muchu B Deng J Peng J Xu Y . E3 ubiquitin ligase RNF180 mediates the ALKBH5/SMARCA5 axis to promote colon inflammation and Th17/Treg imbalance in ulcerative colitis mice. Arch Pharm Res. (2024) 47:645–58. doi: 10.1007/s12272-024-01507-z. PMID: 39060657

[62] Guan Z Wang X Xu C Lu R . ALKBH5 alleviates lower extremity arteriosclerosis by regulating ITGB1 demethylation and influencing macrophage polarization. Heliyon. (2024) 11:e41495. doi: 10.1016/j.heliyon.2024.e41495 39866442 PMC11757785

[63] Strick A von Hagen F Gundert L Klümper N Tolkach Y Schmidt D . The N^6^-methyladenosine (m^6^A) erasers alkylation repair homologue 5 (ALKBH5) and fat mass and obesity-associated protein (FTO) are prognostic biomarkers in patients with clear cell renal carcinoma. BJU Int. (2020) 125:617–24. doi: 10.1111/bju.15019. PMID: 31985880

[64] Xiong J He J Zhu J Pan J Liao W Ye H . Lactylation-driven METTL3-mediated RNA m6A modification promotes immunosuppression of tumor-infiltrating myeloid cells. Mol Cell. (2022) 82:1660–1677.e10. doi: 10.1016/j.molcel.2022.02.033. PMID: 35320754

[65] Sun N Wu Y Liu B Xu P Shen G Li J . YTHDF1 mediates KLF2/VSIG4 axis to regulate kupffer cell polarization to alleviate sepsis-induced liver injury. Genes Immun. (2025). doi: 10.1038/s41435-025-00367-x. PMID: 41198930

[66] Liu J Gao M Xu S Chen Y Wu K Liu H . YTHDF2/3 are required for somatic reprogramming through different RNA deadenylation pathways. Cell Rep. (2020) 32:108120. doi: 10.1016/j.celrep.2020.108120. PMID: 32905781

[67] Feng ZW Yang CF Xiao HF Yuan F Chen F Zhang B . YTHDC1 regulates the migration, invasion, proliferation, and apoptosis of rheumatoid fibroblast-like synoviocytes. Front Immunol. (2024) 15:1440398. doi: 10.3389/fimmu.2024.1440398. PMID: 39534605 PMC11554466

[68] Zhang M Lu N Guo XY Li HJ Guo Y Lu L . Influences of the lncRNA TUG1-miRNA-34a-5p network on fibroblast-like synoviocytes (FLSs) dysfunction in rheumatoid arthritis through targeting the lactate dehydrogenase A (LDHA). J Clin Lab Anal. (2021) 35:e23969. doi: 10.1002/jcla.23969. PMID: 34403518 PMC8418480

[69] Waickman AT Powell JD . mTOR, metabolism, and the regulation of T-cell differentiation and function. Immunol Rev. (2012) 249:43–58. doi: 10.1111/j.1600-065X.2012.01152.x 22889214 PMC3419491

[70] Chapman NM Boothby MR Chi H . Metabolic coordination of T cell quiescence and activation. Nat Rev Immunol. (2020) 20:55–70. doi: 10.1038/s41577-019-0203-y. PMID: 31406325

[71] Akhter S Tasnim FM Islam MN Rauf A Mitra S Emran TB . Role of Th17 and IL-17 cytokines on inflammatory and auto-immune diseases. Curr Pharm Des. (2023) 29:2078–90. doi: 10.2174/1381612829666230904150808. PMID: 37670700

[72] Qin Y Cai ML Jin HZ Huang W Zhu C Bozec A . Age-associated B cells contribute to the pathogenesis of rheumatoid arthritis by inducing activation of fibroblast-like synoviocytes via TNF-α-mediated ERK1/2 and JAK-STAT1 pathways. Ann Rheum Dis. (2022) 81:1504–14. doi: 10.1136/ard-2022-222605. PMID: 35760450

[73] Zheng D Hu J Mai C Huang L Zhou H Yu L . Liver X receptor inverse agonist SR9243 attenuates rheumatoid arthritis via modulating glycolytic metabolism of macrophages. Acta Pharmacol Sin. (2024) 45:2354–65. doi: 10.1038/s41401-024-01315-7. PMID: 38987388 PMC11489696

[74] Vander Heiden MG Cantley LC Thompson CB . Understanding the Warburg effect: the metabolic requirements of cell proliferation. Science. (2009) 324:1029–33. doi: 10.1126/science. PMID: 19460998 PMC2849637

[75] Li Q Chen Y Liu H Tian Y Yin G Xie Q . Targeting glycolytic pathway in fibroblast-like synoviocytes for rheumatoid arthritis therapy: challenges and opportunities. Inflammation Res. (2023) 72:2155–67. doi: 10.1007/s00011-023-01807-y. PMID: 37940690

[76] Weyand CM Goronzy JJ . Immunometabolism in the development of rheumatoid arthritis. Immunol Rev. (2020) 294:177–87. doi: 10.1111/imr.12838. PMID: 31984519 PMC7047523

[77] Napolitano M Bravo E . Lipid metabolism and TNF-alpha secretion in response to dietary sterols in human monocyte derived macrophages. Eur J Clin Invest. (2005) 35:482–90. doi: 10.1111/j.1365-2362.2005.01523.x 16101668

[78] Li R Wu X Peng S Shen J Cheng Y Chu Q . CCR2 antagonist represses fibroblast-like synoviocyte-mediated inflammation in patients with rheumatoid arthritis. Int Immunopharmacol. (2023) 122:110570. doi: 10.1016/j.intimp.2023.110570. PMID: 37390649

[79] Anand PK . From fat to fire: The lipid-inflammasome connection. Immunol Rev. (2025) 329:e13403. doi: 10.1111/imr.13403. PMID: 39327931 PMC11744241

[80] Ouyang B Wu Q Liang J Cao H Xiao J . Polyphenols-mediated immune regulation: Metabolite-driven epigenetic regulatory mechanisms. Phytomedicine. (2025) 149:157535. doi: 10.1016/j.phymed.2025.157535. PMID: 41253023

[81] Shapiro H Thaiss CA Levy M Elinav E . The cross talk between microbiota and the immune system: metabolites take center stage. Curr Opin Immunol. (2014) 30:54–62. doi: 10.1016/j.coi.2014.07.003. PMID: 25064714

[82] Xu S Chaudhary O Rodríguez-Morales P Sun X Chen D Zappasodi R . Uptake of oxidized lipids by the scavenger receptor CD36 promotes lipid peroxidation and dysfunction in CD8+ T cells in tumors. Immunity. (2021) 54:1561–1577.e7. doi: 10.1016/j.immuni.2021.05.003. PMID: 34102100 PMC9273026

[83] Tian X Chen J Hong Y Cao Y Xiao J Zhu Y . Exploring the role of macrophages and their associated structures in rheumatoid arthritis. J Innate Immun. (2025) 17:95–111. doi: 10.1159/000543444. PMID: 39938504 PMC11820663

[84] Chen X Yu C Liu X Liu B Wu X Wu J . Intracellular galectin-3 is a lipopolysaccharide sensor that promotes glycolysis through mTORC1 activation. Nat Commun. (2022) 13:7578. doi: 10.1038/s41467-022-35334-x. PMID: 36481721 PMC9732310

[85] Torres A Pedersen B Cobo I Ai R Coras R Murillo-Saich J . Epigenetic regulation of nutrient transporters in rheumatoid arthritis fibroblast-like synoviocytes. Arthritis Rheumatol. (2022) 74:1159–71. doi: 10.1002/art.42077. PMID: 35128827 PMC9246826

[86] Zhang M Lu N Li HJ Guo XY Lu L Guo Y . Inhibition of lncRNA NEAT1 induces dysfunction of fibroblast-like synoviocytes in rheumatoid arthritis via miRNA-338-3p-mediated regulation of glutamine metabolism. J Orthop Surg Res. (2022) 17:401. doi: 10.1186/s13018-022-03295-y. PMID: 36050752 PMC9438172

[87] Takahashi S Saegusa J Sendo S Okano T Akashi K Irino Y . Glutaminase 1 plays a key role in the cell growth of fibroblast-like synoviocytes in rheumatoid arthritis. Arthritis Res Ther. (2017) 19:76. doi: 10.1186/s13075-017-1283-3. PMID: 28399896 PMC5387190

[88] German HM Ciapaite J Verhoeven-Duif NM Jans JJM . Anaplerosis by medium-chain fatty acids through complex interplay with glucose and glutamine metabolism. J Biol Chem. (2025) 301:108307. doi: 10.1016/j.jbc.2025.108307. PMID: 39955061 PMC12020922

[89] Gao P Tchernyshyov I Chang TC Lee YS Kita K Ochi T . c-myc suppression of miR-23a/b enhances mitochondrial glutaminase expression and glutamine metabolism. Nature. (2009) 458:762–5. doi: 10.1038/nature07823. PMID: 19219026 PMC2729443

[90] Wu J Feng Z Chen L Li Y Bian H Geng J . TNF antagonist sensitizes synovial fibroblasts to ferroptotic cell death in collagen-induced arthritis mouse models. Nat Commun. (2022) 13:676. doi: 10.1038/s41467-021-27948-4. PMID: 35115492 PMC8813949

[91] Wang H Hu X Huang M Liu J Gu Y Ma L . Mettl3-mediated mRNA m6A methylation promotes dendritic cell activation. Nat Commun. (2019) 10:1898. doi: 10.1038/s41467-019-09903-6. PMID: 31015515 PMC6478715

[92] Yao Y Yang Y Guo W Xu L You M Zhang YC . METTL3-dependent m6A modification programs T follicular helper cell differentiation. Nat Commun. (2021) 12:1333. doi: 10.1038/s41467-021-21594-6. PMID: 33637761 PMC7910450

[93] Zhu Y Zhao Y Zou L Zhang D Aki D Liu YC . The E3 ligase VHL promotes follicular helper T cell differentiation via glycolytic-epigenetic control. J Exp Med. (2019) 216:1664–81. doi: 10.1084/jem.20190337. PMID: 31123085 PMC6605754

[94] Wang Y Li L Li J Zhao B Huang G Li X . The emerging role of m6A modification in regulating the immune system and autoimmune diseases. Front Cell Dev Biol. (2021) 9:755691. doi: 10.3389/fcell.2021.755691. PMID: 34869344 PMC8635162

[95] Mobet Y Liu X Liu T Yu J Yi P . Interplay between m(6)A RNA methylation and regulation of metabolism in cancer. Front Cell Dev Biol. (2022) 10:813581. doi: 10.3389/fcell.2022.813581. PMID: 35186927 PMC8851358

[96] Jia Y Li R Huang L Wu X Zhao L Yang H . The glycolysis-HIF-1α axis induces IL-1β of macrophages in rheumatoid arthritis. Arthritis Res Ther. (2025) 27:180. doi: 10.1186/s13075-025-03647-z 41013704 PMC12465665

[97] Wu J Liu L Xu B Wang R Ma W Deng J . FTO enhances OSCC progression via m6A-dependent stabilization of PKM2 mRNA through YTHDF2 modulation. Head Face Med. (2025) 21:21. doi: 10.1186/s13005-025-00431-8 41121381 PMC12542365

[98] Zamudio-Martínez E Delgado-Bellido D Borrego-Pérez J Garcia-Diaz A Herrera-Campos AB Rodríguez-Vargas JM . Tankyrases modulate the hypoxia response through non-catalytic mechanisms affecting HIF-1α. Cell Commun Signal. (2025) 23:477. doi: 10.1186/s12964-025-02480-w. PMID: 41194181 PMC12587615

[99] Jia Y Li R Huang L Wu X Zhao L Yang H . The Glycolysis-HIF-1α axis induces IL-1β of macrophages in rheumatoid arthritis. Arthritis Res Ther. (2025) 27:180. doi: 10.1007/978-981-97-7640-5_110 41013704 PMC12465665

[100] Tian Z Zhang R Ma Y Li J Li Y Lv L . D-mannose suppresses HIF-1α mediated metabolic reprogramming in clear cell renal cell carcinoma. Discov Oncol. (2025) 16:1934. doi: 10.1007/s12672-025-03695-6. PMID: 41118022 PMC12540231

[101] Wang Y Wang Y Gu J Su T Gu X Feng Y . The role of RNA m6A methylation in lipid metabolism. Front Endocrinol. (2022) 13:866116. doi: 10.3389/fendo.2022.866116. PMID: 36157445 PMC9492936

[102] Sun M Yue Y Wang X Feng H Qin Y Chen M . ALKBH5-mediated upregulation of CPT1A promotes macrophage fatty acid metabolism and M2 macrophage polarization, facilitating Malignant progression of colorectal cancer. Exp Cell Res. (2024) 437:113994. doi: 10.1016/j.yexcr.2024.113994. PMID: 38479704

[103] Su Z Lan J Wang Y Ma N Yang J Liang D . Lactylation-driven ALKBH5 diminishes macrophage NLRP3 inflammasome activation in patients with G6PT deficiency. J Allergy Clin Immunol. (2025) 155:1783–99.e8. doi: 10.1016/j.jaci.2025.01.028. PMID: 39900266

[104] Jiao Y Wang Z Diao W Geng Q Wang X Cao X . Increased alleviation of bone destruction in individuals with rheumatoid arthritis via the coinhibition of the METTL3 and YTHDF1 axis by the combination of triptolide and medicarpin. Engineering. (2025) 48:277–91. doi: 10.1016/j.eng.2025.03.014. PMID: 38826717

[105] Gong XP Li W Wu XH Mao LR . METTL3-mediated m6A modification promotes pri-miR-221 maturation to regulate macrophage M1/M2 polarization and immune inflammation in rheumatoid arthritis. Clin Exp Pharmacol Physiol. (2025) 53:e70099. doi: 10.1111/1440-1681.70099. PMID: 41424004

[106] Kim GW Lee DH Jeon YH Yoo J Kim SY Lee SW . Glutamine synthetase as a therapeutic target for cancer treatment. Int J Mol Sci. (2021) 22:1701. doi: 10.3390/ijms22041701. PMID: 33567690 PMC7915753

[107] Olson O Zhang R Proven M Sarmago M Swann J Lowry W . 3007 – GLUTAMINOLYSIS FUELS EMERGENCY MYELOPOIESIS. Exp Hematol. (2023) 124:S54. doi: 10.1016/j.exphem.2023.06.114. PMID: 38826717

[108] Xiao S Duan S Caligiuri MA Ma S Yu J . YTHDF2: a key RNA reader and antitumor target. Trends Immunol. (2025) 46:485–98. doi: 10.1016/j.it.2025.04.003. PMID: 40399203 PMC13715896

[109] Yao F Xu C Gao Y Fu B Zhang L Guo Y . Expression and clinical significance of the m6A reader YTHDF2 in peripheral blood mononuclear cells from rheumatoid arthritis patients. J Immunotoxicol. (2022) 19:53–60. doi: 10.1080/1547691X.2022.2067916. PMID: 35776431

[110] Hao WY Lou Y Hu GY Qian CY Liang WR Zhao J . RNA m6A reader YTHDF1 facilitates inflammation via enhancing NLRP3 translation. Biochem Biophys Res Commun. (2022) 616:76–81. doi: 10.1016/j.bbrc.2022.05.076. PMID: 35649302

[111] Zhao X Peng Y Wang M Tan Q . Methylation of PRDX3 expression alleviate ferroptosis and oxidative stress in patients with osteoarthritis cartilage injury. Arch Rheumatol. (2025) 40:197–210. doi: 10.5152/ArchRheumatol.2025.11031. PMID: 40757971 PMC12260457

[112] He Y Wang W Luo P Wang Y He Z Dong W . Mettl3 regulates hypertrophic differentiation of chondrocytes through modulating Dmp1 mRNA via Ythdf1-mediated m6A modification. Bone. (2022) 164:116522. doi: 10.1016/j.bone.2022.116522. PMID: 35981698

[113] Zhang R Chen P Wang Y Zeng Z Yang H Li M . Targeting METTL3 enhances the chemosensitivity of non-small cell lung cancer cells by decreasing ABCC2 expression in an m^6^ a-YTHDF1-dependent manner. Int J Biol Sci. (2024) 20:4750–66. doi: 10.7150/ijbs.97425. PMID: 39309428 PMC11414383

[114] Li D Cai L Meng R Feng Z Xu Q . METTL3 modulates osteoclast differentiation and function by controlling RNA stability and nuclear export. Int J Mol Sci. (2020) 21:1660. doi: 10.3390/ijms21051660. PMID: 32121289 PMC7084668

[115] Li D He J Fang C Zhang Y He M Zhang Z . METTL3 regulates osteoclast biological behaviors via iNOS/NO-mediated mitochondrial dysfunction in inflammatory conditions. Int J Mol Sci. (2023) 24:1403. doi: 10.3390/ijms24021403. PMID: 36674918 PMC9862541

[116] Hu Y Zhao X . Role of m6A in osteoporosis, arthritis and osteosarcoma (Review). Exp Ther Med. (2021) 22:926. doi: 10.3892/etm.2021.10358. PMID: 34306195 PMC8281110

[117] Xi JF Liu BD Tang GR Ren ZH Chen HX Lan YL . m6A modification regulates cell proliferation via reprogramming the balance between glycolysis and pentose phosphate pathway. Commun Biol. (2025) 8:496. doi: 10.1038/s42003-025-07937-9. PMID: 40140553 PMC11947274

[118] Zheng Y Wei K Jiang P Zhao J Shan Y Shi Y . Macrophage polarization in rheumatoid arthritis: signaling pathways, metabolic reprogramming, and crosstalk with synovial fibroblasts. Front Immunol. (2024) 15:1394108. doi: 10.3389/fimmu.2024.1394108. PMID: 38799455 PMC11116671

[119] Cronstein BN Aune TM . Methotrexate and its mechanisms of action in inflammatory arthritis. Nat Rev Rheumatol. (2020) 16:145–54. doi: 10.1038/s41584-020-0373-9. PMID: 32066940

[120] Flaherty JN Sivasudhan E Tegowski M Xing Z McGinnis MM Hunter OV . The catalytic efficiency of METTL16 affects cellular processes by governing the intracellular S-adenosylmethionine setpoint. Cell Rep. (2025) 44:115966. doi: 10.1016/j.celrep.2025.115966. PMID: 40644296 PMC12330366

[121] Luo Q Lan M Wu Z Wang S Fu P Xiao Q . Decreased ALKBH5 in neutrophil correlates with disease activity in rheumatoid arthritis and ALKBH5 modulates neutrophil autophagy. Sci Rep. (2025) 15:37880. doi: 10.1038/s41598-025-21727-7. PMID: 41162497 PMC12572368

[122] Zhao J Li Y Zhang R Shan Y Song C Cheng Y . Therapeutic gene targets and epigenetic modifications in rheumatoid arthritis: insights from MTX, JAK inhibitors, and LLDT-8. Biochem Biophys Rep. (2025) 42:102038. doi: 10.1016/j.bbrep.2025.102038. PMID: 40417081 PMC12099461

[123] Wang Q Guo X Li L Gao Z Su X Ji M . N^6^-methyladenosine METTL3 promotes cervical cancer tumorigenesis and warburg effect through YTHDF1/HK2 modification. Cell Death Dis. (2020) 11:911. doi: 10.1038/s41419-020-03071-y. PMID: 33099572 PMC7585578

[124] Chen M Wei L Law CT Tsang FH Shen J Cheng CL . RNA N6-methyladenosine methyltransferase-like 3 promotes liver cancer progression through YTHDF2-dependent posttranscriptional silencing of SOCS2. Hepatology. (2018) 67:2254–2270. doi: 10.1002/hep.29683 29171881

[125] Wang Y Zhao M Cui J Wu X Li Y Wu W . Ochratoxin A induces reprogramming of glucose metabolism by switching energy metabolism from oxidative phosphorylation to glycolysis in human gastric epithelium GES-1 cells *in vitro*. Toxicol Lett. (2020) 333:232–41. doi: 10.1016/j.toxlet.2020.08.008. PMID: 32835834

[126] Du W Huang Y Chen X Deng Y Sun Y Yang H . Discovery of a PROTAC degrader for METTL3-METTL14 complex. Cell Chem Biol. (2024) 31:177–83.e17. doi: 10.1016/j.chembiol.2023.12.009. PMID: 38194973

[127] Ahmed S Mahony CB Torres A Murillo-Saich J Kemble S Cedeno M . Dual inhibition of glycolysis and glutaminolysis for synergistic therapy of rheumatoid arthritis. Arthritis Res Ther. (2023) 25:176. doi: 10.1186/s13075-023-03161-0. PMID: 37730663 PMC10510293

[128] Liang S Wan L Wang S Zhang M Wang Y Min W . Crossing the metabolic homeostasis divide: panoramic decoding of therapeutic targets for metabolic-inflammatory crosstalk in rheumatoid arthritis. Front Immunol. (2025) 16:1633752. doi: 10.3389/fimmu.2025.1633752. PMID: 41019065 PMC12460257

[129] Jia N Gao Y Li M Liang Y Li Y Lin Y . Metabolic reprogramming of proinflammatory macrophages by target delivered roburic acid effectively ameliorates rheumatoid arthritis symptoms. Sig Transduct Target Ther. (2023) 8:280. doi: 10.1038/s41392-023-01499-0. PMID: 37500654 PMC10374631

[130] Qiu D Yan B Xue H Xu Z Tan G Liu Y . Perspectives of exosomal ncRNAs in the treatment of bone metabolic diseases: Focusing on osteoporosis, osteoarthritis, and rheumatoid arthritis. Exp Cell Res. (2025) 446:114457. doi: 10.1016/j.yexcr.2025.114457. PMID: 39986599

[131] Jiang X Shi L Feng H Zhang Y Dong J Shen Z . Engineered exosomes loaded with triptolide: an innovative approach to enhance therapeutic efficacy in rheumatoid arthritis. Int Immunopharmacol. (2024) 129:111677. doi: 10.1016/j.intimp.2024.111677. PMID: 38350355

[132] Zhou Z Wu S Li Y Shao P Jiang J . Ginsenoside Rh2-pretreated mesenchymal stem cell exosomes ameliorate collagen-induced arthritis via N6-methyladenosine methylation. Biomater Res. (2025) 29:220. doi: 10.34133/bmr.0220. PMID: 40502627 PMC12153209

[133] Sun X Meng X Piao Y Dong S Dong Q . METTL3 promotes the osteogenic differentiation of human periodontal ligament cells by increasing YAP activity via IGF2BP1 and YTHDF1-mediated m6A modification. J Periodontal Res. (2024) 59:1017–30. doi: 10.1111/jre.13297. PMID: 38838034

[134] Zewail M Gaafar PME Abbas H Elsheikh MA . Innovative rheumatoid arthritis management using injection replacement approach via dual therapeutic effects of hyalurosomes-encapsulated luteolin and dexamethasone. Colloids Surf B Biointerfaces. (2025) 249:114497. doi: 10.1016/j.colsurfb.2025.114497. PMID: 39799610

[135] Li Y Duan Q Huang J Zhao P Cai K . Advances in injectable drug delivery systems for the treatment of rheumatoid arthritis. Biomater Transl. (2025) 6:40–54. doi: 10.12336/biomatertransl.2025.01.004. PMID: 40313572 PMC12041806

[136] Mahmoud DE Kaabachi W Sassi N Tarhouni L Rekik S Jemmali S . The synovial fluid fibroblast-like synoviocyte: a long-neglected piece in the puzzle of rheumatoid arthritis pathogenesis. Front Immunol. (2022) 13:942417. doi: 10.3389/fimmu.2022.942417. PMID: 35990693 PMC9388825

